# Embracing Technology to Develop a Clinical Pharmacology and Medication Safety Elective in Undergraduate Medical Education

**DOI:** 10.1007/s40670-024-02165-y

**Published:** 2024-09-26

**Authors:** William T. Wightkin, Elizabeth B. Graham, Brooke Hooper, Uzoma Ikonne

**Affiliations:** 1 Department of Biomedical and Translational Sciences, Eastern Virginia Medical School, 700 W. Olney Rd., Norfolk, VA 23507 USA; 2 Eastern Virginia Medical School, 825 Fairfax Ave, Norfolk, VA 23507 USA; 3 Department of Internal Medicine, Eastern Virginia Medical School, Box 1980 Norfolk, Norfolk, VA 23501 USA; 4 Fine Family Academy of Medical Educators, Eastern Virginia Medical School, P.O. Box 1980, Norfolk, VA 23501 USA

**Keywords:** Medical education, Pharmacology, Medication safety, Technology

## Abstract

A clinical pharmacology and medication safety elective was developed for fourth-year medical students to enhance students’ foundational pharmacology knowledge and the importance of preventing medication errors. Using video conferencing technology represents a modern approach to facilitate vertical integration of pharmacology curricula and increase multi-institutional and interprofessional collaboration to improve student learning.

Medication errors are associated with extensive financial costs and human harm [1]. Meanwhile, many medical schools are modifying their curriculum to increase clinical exposure to students in the pre-clinical phase restricting the instructional time for pharmacology while the number of new drugs to teach students continues to increase [2]. These findings suggest that students could benefit from more exposure to medical pharmacology before entering medication orders. Medical pharmacology is a broad and complex field of study and limiting medical school pharmacology instruction to only the pre-clinical phase may lead to minimal or no exposure to the clinical application of major topics in pharmacology including medication safety. Applying the approach of the spiral curriculum to pharmacology medical student instruction, where pharmacology topics are revisited to deepen understanding with successive exposure to the topic [3], could solidify and enhance the pharmacology principles learned in the pre-clinical phase. Therefore, offering a fourth-year medical school elective covering core topics in patient and medication safety as well as the safe and cost-effective use of common medications is a strategy that can address these educational concerns.

The COVID-19 pandemic made it necessary for many medical schools to shift to virtual platforms to teach didactic sessions and facilitate online group learning. The use of virtual platforms allowed educators from multiple institutions and professional backgrounds to collaborate and develop new curricula. We used Zoom to collaborate, develop, and implement a 4-week, virtual elective for fourth-year medical students (M4) to expose them to the application of clinical pharmacology principles with pharmacists, physicians, and basic science faculty across three institutions in the spring of 2023. The elective was initially offered to 10 students. All course content was posted to a web-based learning management system and Zoom was used for video-conferenced meetings. Students worked asynchronously viewing videos, reading scientific articles, and working on assessments. Topics included the basics of clinical pharmacology, such as clinically applied pharmacokinetics, drug interactions, renal and hepatic dosing, and the use of drugs in geriatric patients. Specific drug groups were then studied including antimicrobials, analgesics, anticoagulants, and drugs for diabetes. The study of core topics in patient and medication safety was also a primary learning objective. Discussions on error science, error investigation, and just culture were included. Additionally, as each drug group was studied, specific medication safety concerns were identified. The elective included remote synchronous sessions with students. Topics from the preceding week were recapped each Monday. Any questions were answered during these meetings. There was an additional meeting scheduled each elective week: local guest pharmacists and physician experts were recruited to facilitate case study discussions on adverse drug reactions and drug interactions, antimicrobial therapy, fluid and electrolyte imbalances, and computerized clinician drug order entry. The elective sessions and learning objectives are reported in Table 1. The elective course was pass/fail based on attendance at synchronous sessions and completion of problem sets and quizzes. The students were given all answers after the completion of these formative assignments.

**Table 1:** Elective content and learning objective: clinical pharmacology and medication safety

*WEEK ONE*	*Session title*	*Primary learning objective(s)*
Asynchronous sessions	Review of pharmacokinetics and pharmacodynamics	Discuss the mathematical and conceptual relationship between dose, volume distribution and plasma concentration From a serum concentration–time curve, determine a drug’s elimination half-life Describe the relationship between a kinetic process, its half-life and the time it takes to be (nearly) completed Explain the concept of drug targets and be able to list several examples of them Define tolerance, tachyphylaxis, desensitization, refractoriness, and downregulation
	Drug dosage forms and routes of administration	Describe the main routes of drug administration as well as their advantages and disadvantages Be able to match a drug’s route of administration to its serum-concentration time curve Discuss the pharmacologic significance if a drug’s route of administration bypasses its first-pass effect
	Analyzing a drug’s full prescribing information	Describe and understand the categories included in a drug’s FDA full prescribing information
	Strategies to avoid harm from drug interactions and adverse drug reactions	List tactics for preventing and identifying drug interactions and adverse drug reactions
	Pharmacology for special populations: patients with renal and hepatic failure; pediatric patients; geriatric patients	Describe the impact of these special patient populations on drug use and drug dosing
	Problem set on pharmacokinetics and pharmacodynamics	Allow the students to apply basic pharmacological principles
	Problem set on renal and hepatic failure drug dosing	Allow the students to apply drug dosing strategies for these special patient populations
	Introduction to Patient Safety	Recognize contributors to unsafe care existing in healthcare List well-identified safety concerns in patient care that can be improved or eliminated to improve patient outcomes Describe proactive, reactive and predictive safety methods
	Introduction to Medication Safety	Describe the impact of medication errors and adverse drug reactions on patient safety Explain why there is an emphasis on preventing harm from high alert medications
Synchronous sessions	Case studies on adverse drug reactions and drug interactions (facilitator: hospital pharmacist)	Discuss how to recognize and prevent drug interactions and adverse drug reactions
WEEK TWO	**Session title**	**Primary learning objective(s)**
Asynchronous sessions	Review of antibacterial drug classes	For the commonly used antibacterial groups (for examples: penicillins and cephalosporins), describe and compare/contrast the various drugs’ mechanisms of action, spectrums of activity, and adverse effects
	Review of current guidelines for treating pneumonia, urinary tract infections, and skin infections	Know the current clinical guidelines for the treatment of these common infectious diseases
	Clinical pharmacology for analgesics (NSAIDs and Opioids)	Know the mechanisms of action and adverse effects of NSAIDs and opioid analgesics Develop strategies for the safe use of these analgesics
	Clinical pharmacology for insulin and other diabetes medications	Know the mechanisms of action and adverse effects of the various drug categories used in the management of diabetes Describe current treatment approaches for type 1 and type 2 diabetes
	Complete problem sets on pain therapy and diabetes treatment; Complete Bug vs. Drug Comparison Chart for Antibacterials	Give the students an opportunity to construct treatment approaches for the management of pain and diabetes Be able to compare the spectrum of activities for various antibacterials via completion of the assigned chart
Synchronous sessions	Recap discussion on Week One topics; Discussion of Patient Safety Just Culture principles	Describe the role of a robust safety culture in assuring optimal patient safety Define just culture and describe the use of a just culture algorithm in analyzing patient safety adverse events
	Case studies on antimicrobial therapy (facilitators: infectious disease pharmacist and physician)	Via presentation and analysis of patient cases, describe the management of common infectious diseases
WEEK THREE	**Session title**	**Primary learning objective(s)**
Asynchronous sessions	Antihypertensives	Know the mechanisms of action and adverse effects of the various drug categories used in the management of hypertension Describe current guidelines regarding the treatment of hypertension
	IV fluid and electrolytes	Be able to construct a rational fluid and electrolyte prescription Describe management of common electrolyte imbalances
	Antithrombotic therapy	Know the mechanisms of action and adverse effects of the various drug categories that affect blood coagulation Describe antithrombotic treatment approaches for acute coronary syndromes, stroke, DVT and pulmonary embolism List antithrombotics with their recommended reversal agents
	Complete problem sets on hypertension management, IV fluid and electrolytes and antithrombotics	Give the students an opportunity to construct treatment approaches for the management of hypertension, fluid and electrolyte imbalances and blood clotting disorders
Synchronous sessions	Discussion on Week Two topics; discussion on proactive safety techniques; discussion on safety grades for hospitals from Leapfrog Survey	Explain the importance of using proactive methods to prevent harm from healthcare hazards Describe what patient safety issues can be learned from the hospital Leapfrog Survey
	Case-based learning: IV Fluids and Electrolyte (Facilitators: emergency department pharmacist and internal medicine physician)	Via presentation and analysis of patient cases, describe the management of common fluid and electrolyte abnormalities
WEEK FOUR	**Session title**	**Primary learning objective(s)**
Asynchronous sessions	Application of the just culture algorithm for a fatal medication error	After reviewing information about a fatal medication error, analyze the case using the just culture algorithm
Synchronous sessions	Controlling the risks of high-alert medications	List high alert medications and outline recommended strategies to avoid harm with their use
	Medication reconciliation case studies	Describe the safety issues with medication reconciliation and list strategies to avoid patient harm from this process
	Case-based learning: medication safety when using the electronic medical record (facilitators: hospital pharmacists from 2 local medical centers)	Via presentation and analysis of patient cases, describe common errors that accompany medication management using the electronic medical record

Five of the 10 students (50%) responded to the post-elective survey. All survey respondents agreed or strongly agreed that the elective: (1) reinforced their previous knowledge of medical pharmacology, (2) increased their level of confidence in prescribing common medications through the use of case studies, (3) convinced them of the importance of reporting medication errors, and (4) increased their understanding of the pharmacist’s role in patient care. All respondents would recommend this elective to other M4 students. In the open response section of the survey, a student referred to the elective as a “perfect review for students heading into residency.”

Overall, the findings from this study suggest that this 4-week virtual elective was effective in helping support vertical integration of clinical pharmacology content in the curriculum. Further, the results of this study suggest the ability of technology to enhance collaboration across institutions and fields to develop learning opportunities for students.

## Data Availability

The datasets generated during or analyzed during the current study are available from the corresponding author on reasonable request.

## Declarations

This project was deemed exempt by the Eastern Virginia Medical School Institutional Review Board (IRB # 22–11-XX-0220).
